# What’s in a name? Lower extremity fracture eponyms (Part 2)

**DOI:** 10.1186/s12245-015-0076-1

**Published:** 2015-07-25

**Authors:** Philip Kin-Wai Wong, Tarek N Hanna, Waqas Shuaib, Stephen M Sanders, Faisal Khosa

**Affiliations:** Department of Radiology and Imaging Sciences, Emory University School of Medicine, 1364 Clifton Road, Atlanta, GA 30322 USA; Division of Emergency Radiology, Department of Radiology and Imaging Sciences, Emory University Hospital Midtown, 550 Peachtree Street NE, Atlanta, GA 30308 USA; Department of Radiology and Imaging Sciences, Emory University Hospital Midtown, 550 Peachtree Street NE, Atlanta, GA 30308 USA; Department of Emergency Medicine, Emory University Hospital, 531 Asbury Circle, Annex Building, Suite N340, Atlanta, GA 30322 USA

**Keywords:** Eponyms, Fractures, Lower extremities, Imaging

## Abstract

Eponymous extremity fractures are commonly encountered in the emergency setting. Correct eponym usage allows rapid, succinct communication of complex injuries. We review both common and less frequently encountered extremity fracture eponyms, focusing on imaging features to identify and differentiate these injuries. We focus on plain radiographic findings, with supporting computed tomography (CT) images. For each injury, important radiologic descriptors are discussed which may need to be communicated to clinicians. Aspects of management and follow-up imaging recommendations are included. This is a two-part review: Part 1 focuses on fracture eponyms of the upper extremity, while Part 2 encompasses fracture eponyms of the lower extremity.

## Introduction

Eponyms are embedded throughout medicine; they can be found in medical literature, textbooks, and even mass media. Their use allows physicians to quickly provide a concise description of a complex injury pattern. Eponymous extremity fractures are commonly encountered in the emergency setting and are frequently used in interactions amongst radiologists, emergency clinicians, and orthopedists. Unfortunately, the imprecise use of eponyms can result in confusion and miscommunication [1]. In this two-part series, our goal is to provide emergency providers with consistent, accurate definitions and depictions of commonly and less frequently encountered extremity fracture eponyms, keying in on important imaging features that differentiate these fractures. We illustrate fundamental descriptors of each injury that a clinician should expect in a radiology report. We also briefly review the mechanism of each injury, associated complications, any follow-up imaging needed, and treatment.

**Fig. 1:**
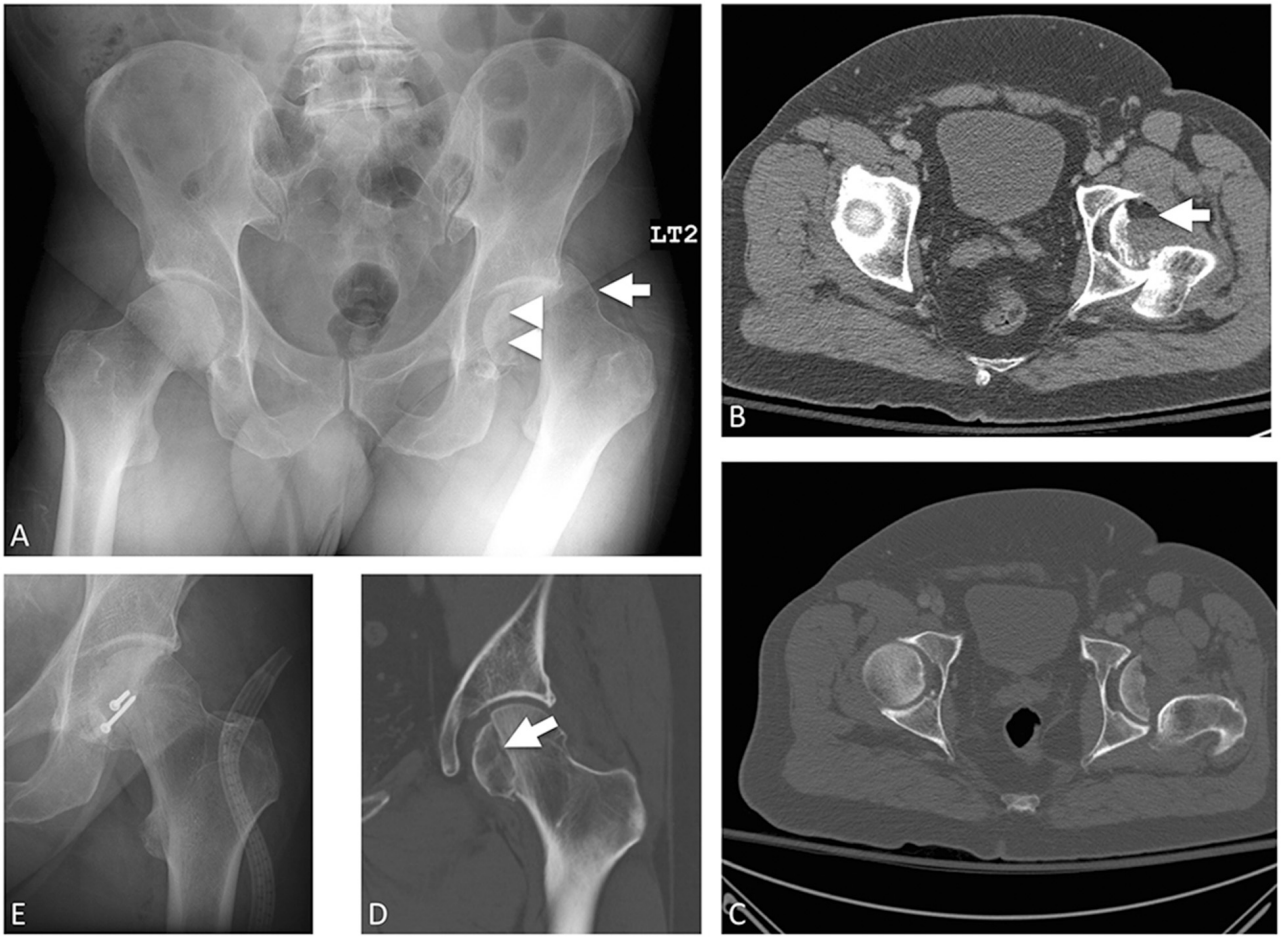
Entire treatment course of a Pipkin type IV femoral head fracture in a patient with posterior hip dislocation status post motor vehicle collision. Initial AP pelvic radiograph (**a**) with posterior superior femoral head dislocation (*arrow*) and femoral head fracture, with a residual femoral head fragment remaining in the acetabulum (*arrowhead*). **b** Axial CT showing lipohemarthrosis, with floating fatty liquid suspending atop pooling blood (*arrow*), resulting from traumatic exposure of the marrow. **c** Bone windows from axial CT. Femoral head fracture and posterior dislocation better demonstrated. **d** Post reduction hip CT coronal. Minimally displaced fracture line visible (*arrow*). **e** Post-operative AP hip radiograph with femoral head screws

**Fig. 2:**
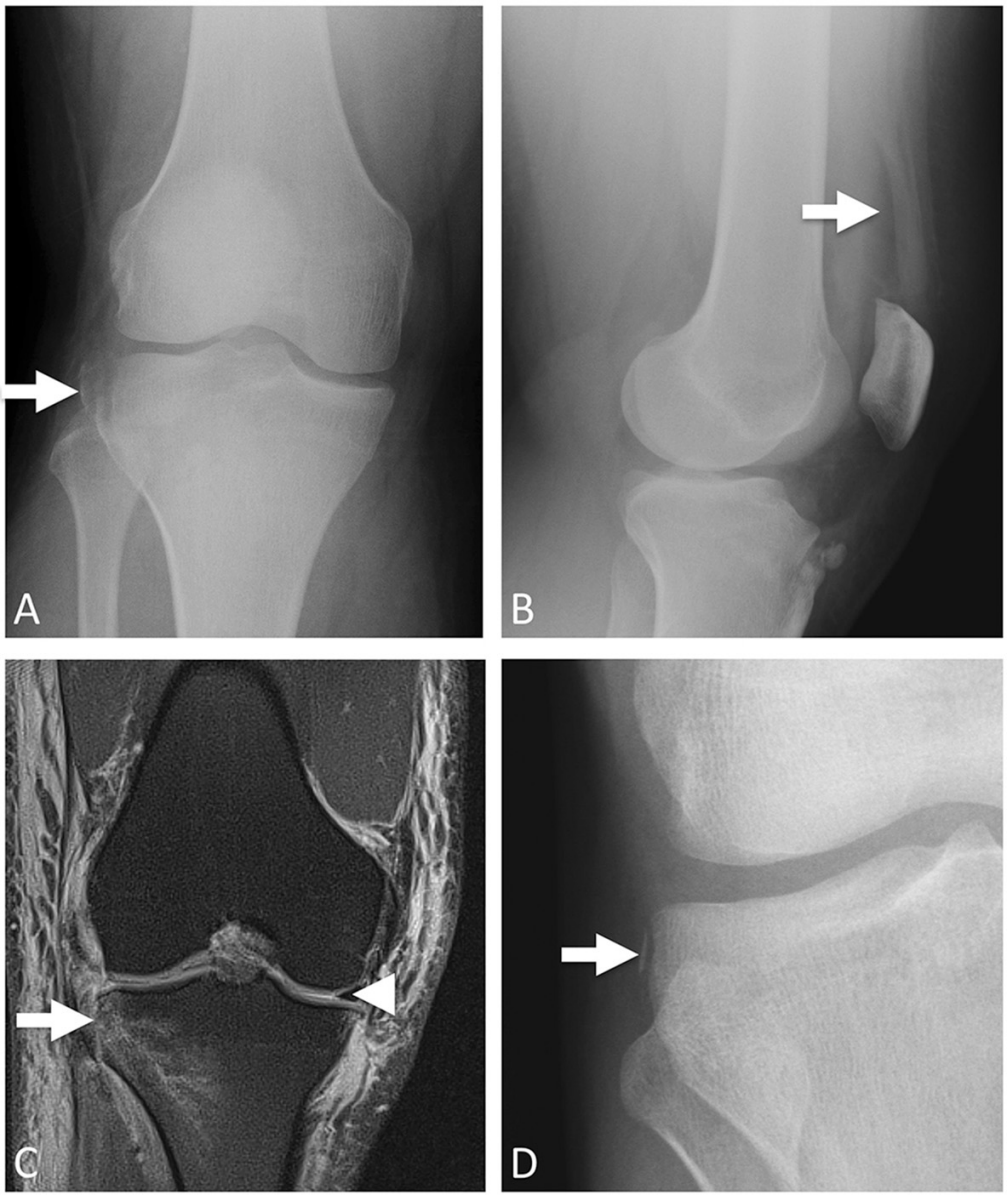
Segond fracture. **a** AP knee radiograph with large Segond fracture (*arrow*). **b** Lateral knee radiograph in the same patient shows a lipohemarthrosis, which confirms fracture-induced exposure of the fatty marrow cavity to the articular space. **c** Coronal proton density (PD) fat saturated images showing the Segond fracture (*arrow*). The bright signal in this image is all edema, since the fat signal is suppressed. The patient had an ACL tear (not shown). Note the presence of the medial meniscus (*arrowhead*) and the absence of the lateral meniscus on the same image, confirming a complete lateral meniscus tear with displacement. **d** Another patient AP knee radiograph. Thin, small Segond fracture (*arrow*). This patient did have an ACL tear; this shows how subtle these fractures can be but still be associated with substantial injury

**Fig. 3:**
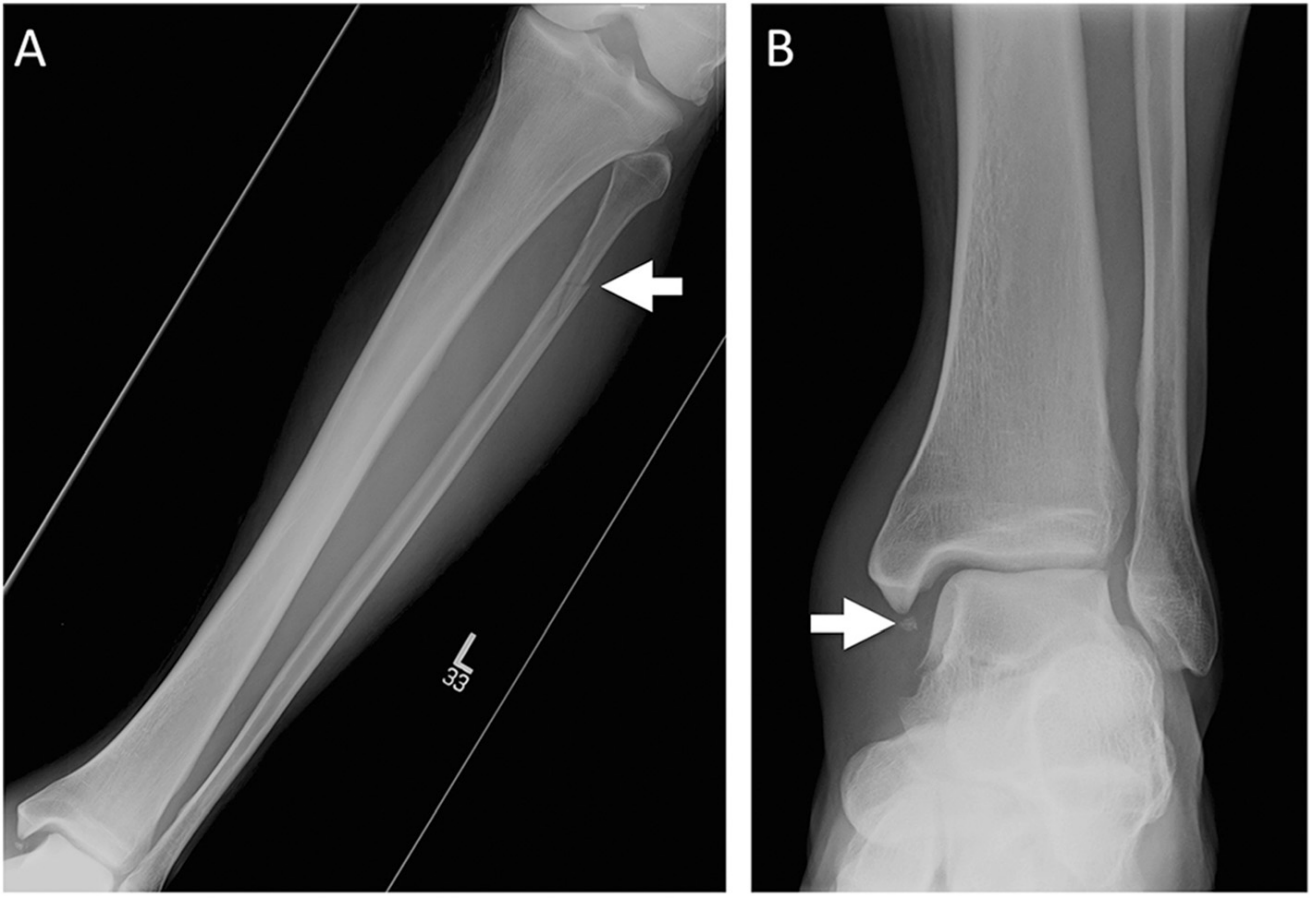
Maisonneuve fracture. **a** AP tibula/fibula radiograph with minimally displaced fracture of the proximal fibular shaft (*arrow*). **b** AP ankle radiograph in the same patient. Soft tissue swelling overlying the lateral malleolus, with avulsion fracture of the inferior aspect of the medial malleolus (*arrow*). This patient had confirmed disruption of the syndesmosis, with subsequent syndesmotic fixation

**Fig. 4:**
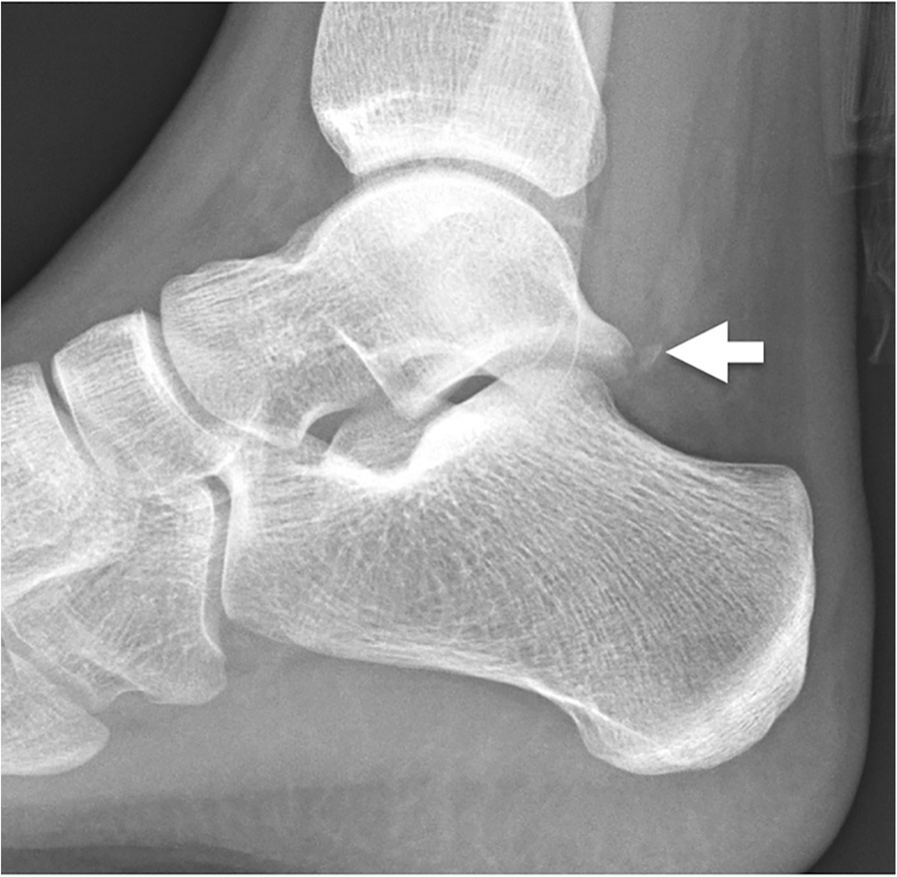
Shepherds fracture. Fracture of the lateral tubercle of the posterior process of the talus (*arrow*)

**Fig. 5:**
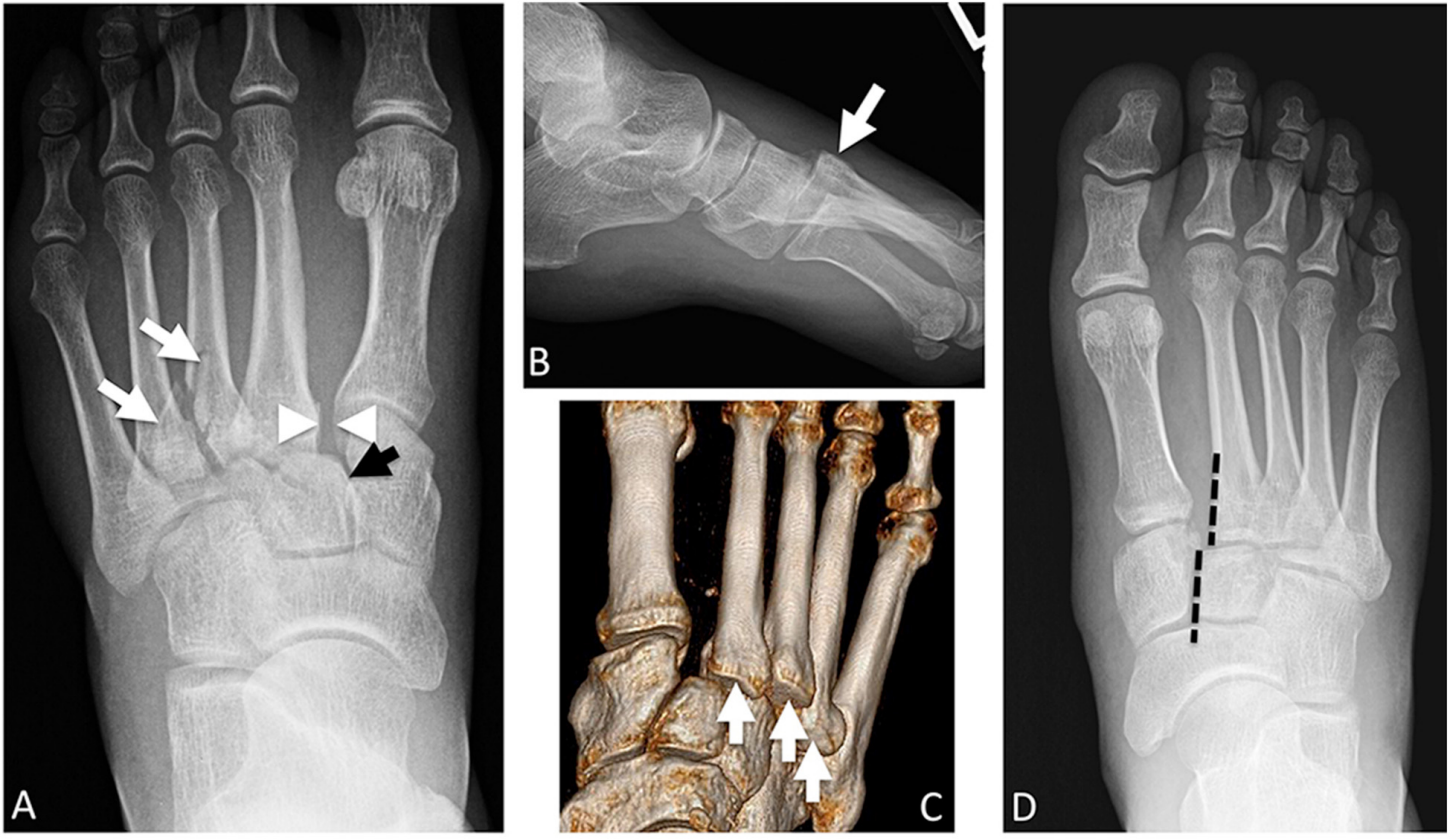
Lisfranc fracture-dislocation. **a** AP view of the foot with widening of the space between the first and second metatarsal bases (*arrowheads*). Misalignment of the second metatarsal base from the middle cuneiform (medial margin of middle cuneiform demarcated with *black arrow*). Fractures of the bases of the third and fourth metacarpals (*white arrows*). **b** Lateral view in the same patient shows dorsal displacement of the second to fourth metatarsal bases (*arrow*). **c** Volume rendered CT reformat in a different patient with dislocation of the second, third, and fourth metatarsal bases. **d** Different patient. Additional Lisfranc fracture dislocation. Here, the misalignment of the second metatarsal base from the middle cuneiform is shown with *dashed lines*—these should be in the same line. Again, there is widening of the first and second metatarsal base interspace

**Fig. 6:**
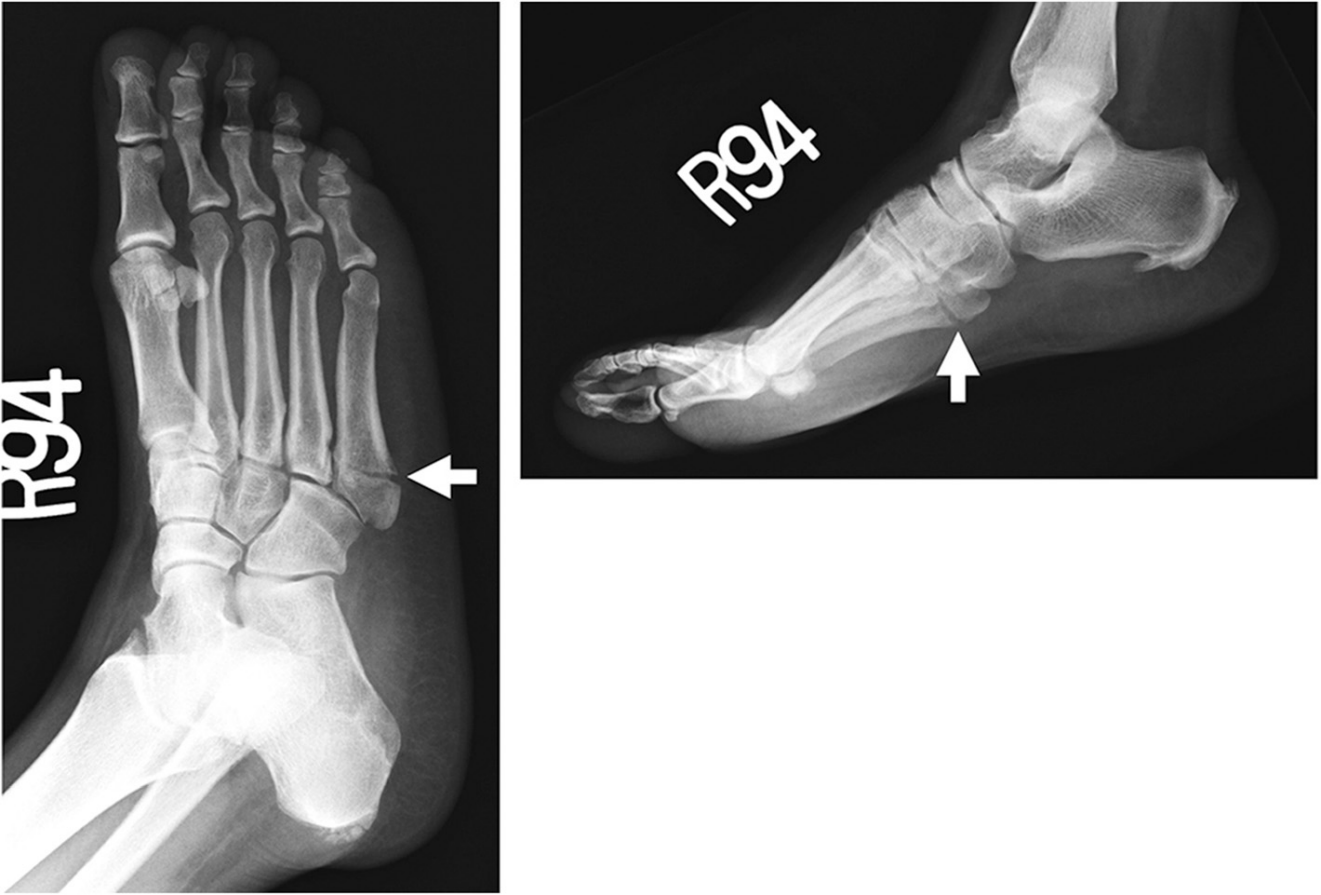
Jones fracture. Transverse fracture 2 cm from the base of the fifth metatarsal

## Review: Lower extremity fracture eponyms

### Pipkin fracture

Femoral head fractures are relatively uncommon and are typically associated with hip dislocations after severe high-impact trauma such as a motor vehicle collision. Femoral head fractures are commonly grouped into the Pipkin classification (see Table 1) after the work of the orthopedic surgeon Garrett Pipkin in 1957 (Fig. 1) [2]. Hip fracture-dislocations are clinical emergencies requiring immediate reduction to prevent osteonecrosis. Anterior-posterior (AP) and lateral radiographs will show posterior dislocation of the femoral head, which appears smaller than the contralateral normal side. If there is suspicion for an acetabular fracture, then Judet oblique views should be obtained. Associated fractures should be identified, with particular attention to the femoral neck [3, 4]. CT of the pelvis should be obtained after closed reduction or prior to open reduction of an irreducible injury in order to better characterize the pattern, degree of comminution, presence of loose bodies, and the congruity of the hip joint [5]. Emergent open reduction is indicated when there is nonanatomic reduction of the femoral head, hip joint instability, or the presence of intraarticular fragments, which prevent joint congruity [3, 4, 6, 7].

**Table 1:** Pipkin classification

Type I	Inferior to the fovea capitis femoris
Type II	Superior to the fovea capitis femoris
Type III	Type I or type II with associated femoral neck fracture
Type IV	Type I or type II with associated acetabular rim fracture

### Segond fracture

First described by the French surgeon Paul Segond in 1879, the Segond fracture may be the best known avulsion fracture of the lower extremity [8]. This eponym refers to a small, vertical avulsion of the proximal lateral tibia just inferior to the tibial plateau Fig. 2. The Segond fracture results from varus stress on an internally rotated knee [9]. AP radiographs are sufficient for diagnosis. The fracture fragment is usually crescentic with approximately 3 mm displacement from the tibial metaphysis [10]. It is important to distinguish the irregular tibial donor site so as to not confuse the Segond fracture with an avulsion fracture of the Gerdy tubercle, which is more anterior and distal and can be distinguished on the lateral radiograph. Although a subtle finding, the Segond fracture is of considerable clinical significance in its extremely high association with tears of the anterior cruciate ligament (ACL) (75–100 % of cases), meniscal tears (66 %), as well as avulsion of the fibular attachment of the long head of the biceps and fibular collateral ligament [11, 12]. Segond fractures should prompt non-emergent MR imaging of the knee [8, 9, 13]. There is more recent recognition of a “reverse Segond fracture,” consisting of a mirror-image crescentic fracture of the medial tibial plateau. This entity has a weaker association with posterior cruciate ligament (PCL) injury and medial meniscus tears and should also prompt non-emergent MR imaging [14]. Treatment involves repair of the associated ligamentous or meniscal tears.

### Maisonneuve fracture

The Maisonneuve fracture, named after the French surgeon Jacque Gilles Maisonneuve, is a spiral fracture of the proximal third of the fibula with associated disruption of the distal tibiofibular syndesmosis Fig. 3 [15]. The fracture results from an injury cascade involving the ankle, where external rotation is applied to a pronated or supinated foot [16]. Rupture of the stabilizing ligaments of the distal tibiofibular syndesmosis will result in widening of the ankle mortise on radiographs. Additional findings such as avulsion fracture of the medial or posterior malleoli, or tear of the deltoid ligaments may also be present [17, 18]. A Maisonneuve fracture implies an unstable ankle, despite normal position of the talus and ankle mortise. It is important to remember that non-weight-bearing views, the ones most often ordered in the emergency department, may not demonstrate widening of the ankle mortise. Maisonneuve fractures may be missed as patient and physicians focus attention on the ankle as the major site of complaint and patients may not complain of pain upon palpation of the proximal fibula [16]. Maisonneuve fractures should be suspected whenever there is lateral talar displacement or tibiofibular widening without distal fibular fracture [19]. In these cases, stress radiographs and full-length tibiofibular radiographs should be obtained [20]. The goal of treatment is to maintain a normal ankle mortise, which usually requires open reduction due to the frequency of ankle instability [17].

### Gosselin fracture

The French surgeon Leon Athanese Gosselin first described the Gosselin fracture [15] as a V-shaped fracture of the distal tibia with extension into the tibial plafond, dividing it into anterior and posterior segments [15]. Distal tibia fractures that involve the articular surface or tibial plafond are also known under the umbrella term “Pilon fractures.” Pilon fractures are quite complex with many variations, usually as a result of axial loading of the weight-bearing surface of the tibia. The degree of comminution, soft tissue swelling, and articular incongruity dictate surgical management which is initially external fixation followed by delayed definitive fixation if the soft tissue swelling is severe [21].

### Pott fracture

The Pott fracture has inappropriately evolved into a term to describe a bimalleolar fracture. Percival Pott originally described it in 1768 as a fracture of the distal fibula, 2–3 in. proximal to the ankle joint, with an associated tear of the deltoid ligaments and lateral displacement of the talus [22–24]. This type of injury results from a direct force resulting in eversion at the ankle [25]. Due to the often incorrect usage of this eponym and the development of newer more detailed ankle fracture classification systems, we suggest this eponym not be used in clinical practice.

### Shepherd fracture

The Shepherd fracture is named after the Canadian surgeon Francis Shepherd and refers to a fracture of the lateral tubercle of the posterior process of the talus Fig. 4 [26], typically resulting from ankle inversion, forced plantar flexion, or direct compression injury in which the posterior talofibular ligament avulses the tubercle [27]. This fracture is best seen on lateral radiographs [28], although the sensitivity of radiographs in recognizing talar fractures is only 78 % [29]. Furthermore, in the setting of trauma, talar and other associated fractures may not be completely identified on plain radiographs; thus, CT should be considered for complete evaluation [29–31]. Of note, Shepherd’s fracture may be mistaken for an os trigonum, an accessory bone from a secondary ossification center posterior to the lateral tubercle, which is a normal finding [27, 28, 32]. Typically, an os trigonum is rounded or oval with smooth corticated edges as opposed to a sharply marginated non-corticated irregular fracture. In equivocal cases, CT, MR, or even a technetium bone scan of the ankle may be helpful [32]. Complications of talar fractures include chronic pain, arthrosis, and rarely avascular necrosis [27]. Treatment of the Shepherd fracture is typically immobilization, although depending on symptoms, delayed excision of the fragments may be necessary [33].

### Tillaux fracture

The Tillaux fracture was described by Sir Astley Cooper in 1822 and further characterized by Paul Tillaux in cadaveric studies in 1845. The Tillaux fracture is an avulsion fracture of the anterolateral tubercle of the distal tibia caused by a pull of the anteroinferior tibiofibular ligament during external rotation [34]. This typically occurs in adolescent patients, as the ligament is usually stronger than the anterolateral epiphysis, which at this time of development, is open and susceptible to injury [35]. The Tillaux fracture is a Salter Harris type III injury and is often apparent on AP, lateral, and mortise conventional radiographic views. CT has been found to have better sensitivity in diagnosing Tillaux fractures as well as detecting fracture displacement greater than 2 mm, which is the indication for open reduction [36–39]. Growth arrest, degenerative arthritis, and ankle instability are feared complications [35, 36, 40].

### Lisfranc fracture

The Lisfranc joint is the tarsometatarsal joint complex which joins the forefoot and midfoot and is named after Jacque Lisfranc de Saint-Martin, a famous French surgeon who performed forefoot disarticulations at this joint [41]. The articulation consists of nine osseous structures: five metatarsals (M1-M5), three cuneiforms (C1-C3), and the cuboid, with further stabilization from a complex arrangement of ligaments. Lisfranc injuries can be subdivided into Lisfranc fracture-displacements due to high-impact injuries versus Lisfranc midfoot sprains due to low-impact injuries. Radiography is the initial imaging study of choice [41]. These fractures may be subtle. A small chip fracture at the M1-M2 interspace, known as the “fleck sign,” may be the only indicator of Lisfranc injury [41–43]. The gap between C1 and M2 should be less than 2 mm [44, 45]. Malalignment or C1-M2 widening suggests Lisfranc injury Fig. 5. Lateral views can show step-offs at the tarsometatarsal joint [46]. Equivocal cases should be further evaluated with weight-bearing or stress radiographs searching for diastasis and step-offs on stress views, that were not seen on resting views [41, 42, 46]. In cases involving a serious mechanism, CT may be beneficial to diagnose or further characterize Lisfranc injuries. MR imaging is recommended in low-grade midfoot sprains due to its superior sensitivity in the detection of ligamentous injuries. Occasionally, when radiography, CT, or MRI are equivocal, bone scintigraphy may show increased radiotracer uptake, suggestive of Lisfranc injuries [41]. Delayed diagnosis may lead to poor outcomes such as arch deformity, chronic pain, or osteoarthritis. For mild Lisfranc injuries with less than 2 mm of diastasis between the first and second metatarsals, nonoperative treatment with immobilization can be pursued. Otherwise, instability or frank dislocation should be treated surgically with either closed reduction under fluoroscopy and fixation with percutaneous screws or open reduction and internal fixation [41, 45].

### Chopart fracture-dislocation

The Chopart joint, also known as the midtarsal or transverse tarsal joint, consists of the calcaneocuboid and talonavicular joints, which join the midfoot and hindfoot. This space was described by the French surgeon Francois Chopart as another potential area for disarticulation [47]. In significant high-energy trauma, these joints may be displaced [48], with associated navicular, cuboid, calcaneal, or talar fractures. This constellation is known as the Chopart fracture-dislocation. Displacement may be in any direction according to the direction of the force [48, 49]. Due to the low sensitivity of radiography in the detection of midfoot fractures, evaluation with CT is recommended, as untreated midfoot fractures often have poor outcomes such as chronic pain, arthritis, and decreased functional ability [50]. Urgent reduction is necessary for treatment of Chopart fracture-dislocations, with subsequent open reduction if anatomical alignment cannot be maintained [50–52].

### Jones fracture

Sir Robert Jones first described his own fracture of the fifth metatarsal, which occurred while dancing, as a transverse fracture at the proximal three-fourth segment of the shaft distal to the styloid Fig. 6 [53, 54]. The Jones fracture should be differentiated from the “Dancer’s fracture,” (or pseudo-Jones fracture), which is an avulsion fracture of the fifth metatarsal base, proximal to the more diaphyseal Jones fracture [13, 55]. The term Jones fracture was later defined as a transverse fracture at the metaphyseal/diaphyseal junction without distal extension beyond the fourth to fifth intermetatarsal articulation [56, 57]. Three views of the foot—AP, lateral, and oblique radiographs—are sufficient for diagnosis of a Jones fracture. Jones fractures take longer to heal than do avulsion fractures and have high rates of nonunion, delayed union, or refracture due to the watershed blood supply [53, 57]. Jones fractures can be treated with non-weight-bearing leg casting versus operative treatment with intramedullary screw fixation.

## Conclusions

Fracture eponyms are frequently used in everyday practice by radiologists, emergency clinicians, and orthopedists. Accurate knowledge of eponymous fractures can facilitate patient care by helping radiologists and emergency clinicians efficiently convey a great deal of information in an extremely concise manner. This concludes our two-part review of eponymous fractures of the extremities. For a brief summary of the reviewed lower extremity fracture eponyms, please see Table 2.

**Table 2:** Lower extremity fracture eponyms

Lower extremity fracture eponyms	Fracture pattern
Pipkin	Femoral head fracture typically associated with hip dislocation. See Table 1 for types.
Segond	Small avulsion fracture of the proximal lateral tibia just inferior to the tibial plateau. High association with ligamentous injury.
Maisonneuve	Spiral fracture of the proximal third of the fibula with associated disruption of the distal tibiofibular syndesmosis
Gosselin	V-shaped intra-articular fracture of the distal tibia
Pott	Fracture of the distal fibula, 2–3 in. proximal to the ankle joint.
Shepherd	Fracture of the lateral tubercle of the posterior talar process
Tillaux	Avulsion fracture of the anterolateral tubercle of the distal tibia
Lisfranc	Fracture-dislocation of the tarsometatarsal joints
Chopart	Fracture-dislocation of the midtarsal joint spaces
Jones	Transverse fracture involving the fifth metatarsal proximal shaft

## Abbreviations

ACL: Anterior cruciate ligament
AP: Anterior-posterior
C1: Medial cuneiform
C2: Middle cuneiform
C3: Lateral cuneiform
CT: Ctomography
M1-M5: First through fifth metatarsals
MRI: Magnetic resonance imaging
PCL: Posterior cruciate ligament
